# The path of no return—Truncated protein N‐termini and current ignorance of their genesis

**DOI:** 10.1002/pmic.201500043

**Published:** 2015-06-15

**Authors:** Nikolaus Fortelny, Paul Pavlidis, Christopher M. Overall

**Affiliations:** ^1^ Department of Biochemistry and Molecular BiologyUniversity of British ColumbiaVancouver, BritishColumbiaCanada; ^2^ Department of Oral Biological and Medical SciencesUniversity of British ColumbiaVancouver, BritishColumbiaCanada; ^3^ Centre for Blood ResearchUniversity of British ColumbiaVancouverBritish ColumbiaCanada; ^4^ Centre for High Throughput BiologyUniversity of British ColumbiaVancouverBritish ColumbiaCanada; ^5^ Department of PsychiatryUniversity of British ColumbiaVancouverBritish ColumbiaCanada

**Keywords:** Alternative translation, Protease cleavage, Protease web, Systems biology, Terminomics, TAILS, TopFIND

## Abstract

Almost all regulatory processes in biology ultimately lead to or originate from modifications of protein function. However, it is unclear to which extent each mechanism of regulation actually affects proteins and thus phenotypes. We assessed the extent of N‐terminal protein truncation in a global analysis of N‐terminomics data and find that most proteins have N‐terminally truncated proteoforms. Because N‐terminomics analyses do not identify the process generating the identified N‐termini, we compared identified termini to the three N‐termini generating events: protein cleavage, alternative translation, and alternative splicing. Of these, we sought to identify the most likely cause of N‐terminal protein truncations in the human proteome. We found that protease cleavage and alternative protein translation are the likely cause for most shortened proteoforms. However, the vast majority (about 90%) of N‐termini remain unexplained by any of these processes identified to date, so revealing large gaps in our knowledge of protein termini and their genesis. Further analysis and annotation of terminomics data is required, to which end we have created the TopFIND database, a major systematic annotation effort for protein termini. We outline the new features in version 3.0 of the updated database and the new bioinformatics tools available and encourage submission of generated data to fill current knowledge gaps.

Protein products of a gene can be largely variable. From 20061 human proteins (neXtProt database 1, release 2015‐01‐01) many more proteoforms are created, which result in millions of different proteins through modifications at the mRNA and the protein level. Whereas post‐transcriptional modification of genomic sequences by RNA splicing is irreversible, commonly considered post‐translational chemical modifications are often reversible modifications to specific amino acid residues, for example, by phosphorylation or acetylation. However, irreversible modifications to protein chains also are now increasingly recognized as playing important roles in generating diversity in protein structure and sequence and hence function and cellular or tissue phenotypes 2.

## 1 Protein truncation as a post‐translational modification

One irreversible modification to proteins involves truncation of proteins to create new, shorter proteoforms with new internal N‐ or C‐termini. Protein truncation has been postulated to have a great impact on generating diversity in the human proteome 2 and to increase the functional repertoire of proteins by precise alteration in the biological properties of proteins 3. Successful terminomics techniques 4, 5, dedicated to the identification of the precise position of all protein termini in tissues in vivo or in cells, consistently discover that about 50% of N‐terminal peptides map internally in proteins with 44% in murine skin 6, 68% in human erythrocytes 7, and 77% in human platelets 8. This unexpectedly high percentage means that many populations of a protein occur that do not start at their canonical genetic encoded N‐termini yet this has largely been overlooked in proteomics data analyses and in their biological interpretation. In addition to the impact on the proteome composition and on the emergent change in functional properties of the altered proteins, a major question is the nature of the mechanism generating N‐termini, one that is especially relevant for designing therapeutics. Protein truncations are generally thought to be the result of protease activity and so proteases may be new drug targets if their substrates are a disease driver. Notwithstanding the pervasiveness of proteolysis in vivo, neo N‐ and C‐termini can also result from alternative translation and alternative splicing events.

Correspondence concerning this and other Viewpoint articles can be accessed on the journals' home page at: http://viewpoint.proteomics‐journal.de

Correspondence for posting on these pages is welcome and can also be submitted at this site.

The assignment of the genesis and impact of terminal peptides on protein function, as well as the importance for the biological system, are a hurdle in current terminomics data analysis that often take significant time. Thus, the TopFIND database was recently updated in January 2015 9 with new data and analysis tools to aid terminomics analyses and assignment of cleavages to the relevant proteases—TopFIND now has 165044 N‐termini and 130182 C‐termini in 90696 proteins from *Homo sapiens*, *Mus musculus*, *Arabidopsis thaliana*, *Saccharomyces cervisiae*, and *Escherichia coli*, thus representing the most comprehensive collection of termini and their evidences.

## 2 The genesis of protein neo‐termini

As the main systematic annotation effort of protein N and C termini data, the knowledgebase TopFIND details a huge amount of evidence for termini derived from four main sources: direct experimental observation of N and C‐termini in terminomics screens (termed here “observed” termini); termini predicted from the biochemical and structural characterization of the protease and its substrates (designated here “cleavage” termini); termini predicted from alternative translation events found by global translation initiation sequencing stored in TISdb 10; and finally termini predicted from alternatively spliced transcripts curated from sequencing data in Ensembl 11. Without direct evidence of their genesis from proteomics analyses we designate termini in the last three categories as “inferred” termini. In this present analysis, we used TopFIND to compare observed N‐termini with inferred N‐termini in order to answer crucial questions about the N‐terminome, in particular, to identify the position of internal N‐termini in proteins and to assess the processes generating the non‐canonical N‐termini.

## 3 Distribution of protein termini

**Figure 1:**
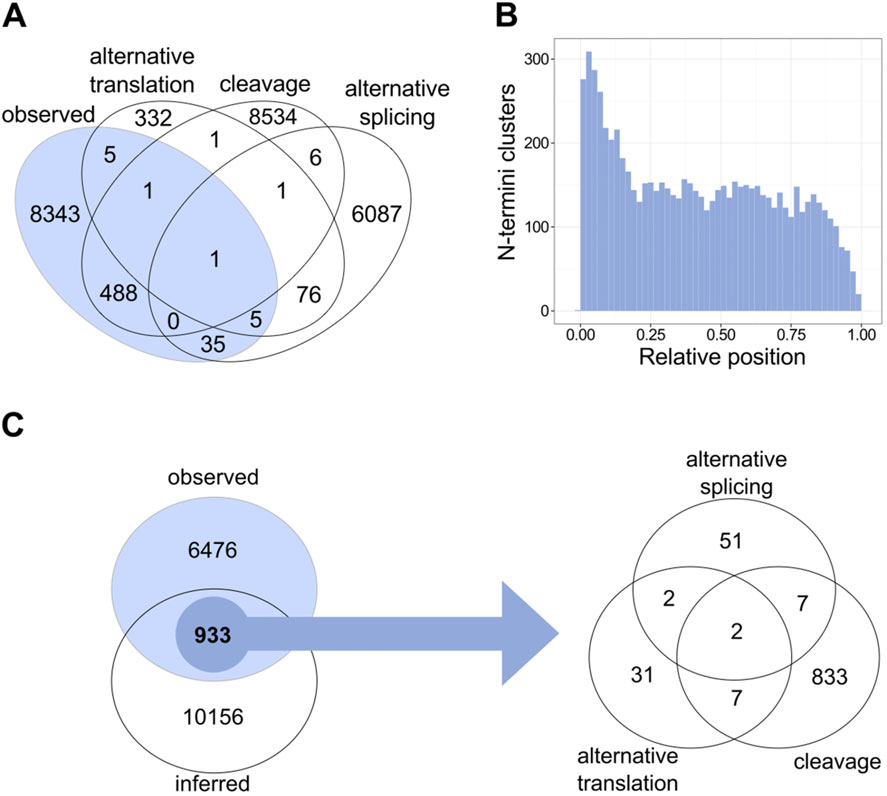
(A) Overlap between observed (blue) and inferred human N‐termini in TopFIND. Inferred N‐termini include those that are predicted from knowledge of sites of cleavage, alternative splicing, or alternative translation. N‐termini are counted by position, i.e. an N‐terminus identified multiple times in different experiments at the same position is only counted once. (B) Position of the 7409 observed and internal N‐termini clusters in proteins relative to the full protein length. (C) Overlap between inferred and observed (blue) N‐termini clusters in TopFIND. The arrow points to the systematic breakdown of the 933 observed and inferred N‐termini clusters to the biological processes generating those N‐termini.

We analyzed human N‐termini observed by terminomics screens and compared these to N‐termini inferred from cleavage events, alternative translation, or alternative transcription as annotated in TopFIND, by counting the overlap between the instances of N‐termini in each group. Counting N‐termini in a position‐specific and not an experiment‐specific manner, we avoided recounting the same N‐terminus multiple times—although this does underestimate cleavage events where two or more proteases cut at the same site—as it is the case when, for example, cleavage is facilitated by protein structural features such as in flexible N or C terminal protein sequences, linker regions between domains, or in exposed loops on or between domains. We identified 48095 observed or inferred N‐termini in the human proteome. Focusing on N‐termini that do not correspond to the canonical start site of the protein (i.e. carboxyl to position > 3 to account for initiator methionine processing plus one exopeptidase or diaminopeptidase event), we identified 23915 observed and inferred N termini as shown in Fig. 1A.

We then collapsed together N‐termini if they are close (distance < 3 amino acids) to avoid recounting the same terminus multiple times that might be due to proteolytic ragging by exopeptidase activity in vivo. Again, this may underestimate the number of distinct termini due to independent cleavage events by the same or different proteases within the 3‐residue cluster. Focusing on observed N‐termini, we identified 9843 N‐termini clusters that contain at least one observed N‐terminus across the full length of the protein. Notably, 7409 of these were internal in the protein (again carboxyl to position > 3 with the remaining 2434 clusters at positions 1–3) and mostly were equally distributed along proteins as shown in Fig. 1B, with a peak observed at or near the original N‐terminus. Thus the clusters mostly did not represent processing of N‐terminal methionine with or without further aminopeptidase or diaminopeptidase activity (only 3.5% of 7409 clusters are between position 3 and 10), under 10% were the consequence of protein maturation to remove signal or transit peptides (9.2% of clusters were between position 9 and 30), and the vast majority (87.2% of clusters beyond position 30) represented the generation of novel shortened proteoforms. A similar distribution but with lower numbers was observed in the original TopFIND publication 12 and has not changed by the addition of newer datasets. We expect that in general these represent true protein N‐termini and not randomly generated peptide fragments, because these peptides were sufficiently stable to accumulate to levels to be reliably identified by mass spectrometry. Moreover, typically, terminomics experimenters report great care to avoid proteolysis at and after sample collection by immediate incorporation of protease inhibitors and maintaining samples on ice or frozen whenever feasible. In summary, our analysis highlights the large percentage of internal termini in the human proteome and shows that examples of new protein starts will be found virtually anywhere in a protein sequence.

## 4 The gap between observed and inferred termini

We next investigated how many observed N‐termini were explained by inferred termini in TopFIND. As described above, inferred N‐termini are predicted from a reported cleavage, an alternative spliced transcript start site, or an alternative translation start site reported at the position in question. An experimentally observed N‐terminus identified at the same position as an inferred terminus was considered to be explained by at least one N‐termini generating process. Of the 7409 internal clusters, only 933 were explained in that they had an associated evidence for an inferred terminus (Fig. 1C). Consequently, for the large majority (87%) of N‐termini clusters there was no explanatory N‐terminus generating biological process reported to be associated with these termini. Thus huge gaps were found in our current knowledge of N‐termini generating processes despite their great impact on the proteome. Further aggravating the situation, we are likely reporting an underestimation, because inferred N‐termini in the vicinity (+/− 3 amino acids) of the observed N‐terminus were taken into account in this analysis. When we only counted inferred N‐termini at the precise position of the observed N‐termini, we were only able to explain 535 of 8878 N‐termini (6%), with 94% of observed N‐termini (8343) remaining unexplained (Fig. 1A).

In the clustered data, most of the 933 explained N‐termini clusters (849) fell close to a protease cleavage site (Fig. 1D). This effect was real and not simply due to annotation biases favoring cleavages. Of 7141 clusters containing cleavage‐inferred N‐termini, 849 (11.9%) were found in actual experimentally observed N‐termini as did 62 (1.7%) of 3590 clusters containing splice site‐inferred N‐termini and 42 (9.9%) of 425 clusters containing N‐termini inferred from alternative translation. Therefore, protein cleavage and alternative translation are the main two mechanisms generating internal protein N‐termini annotated to date. Protease activity and alternative translation are also likely candidates to largely explain the remaining 6476 observed clusters, because the search space for cleavage and alternative translation 13, 14 remains largely unexplored. Indeed, substrate annotation is only available for about half of all human proteases and even for those proteases, their substrate repertoires remain mostly unexplored 15. This is partly due to study biases 16, where few proteases are well studied and many ignored, but it also reflects the lack of database annotation of known cleavages. Hence the urgent unmet need for the community to upload experimental data to the appropriate databases including MEROPS 17 and TopFIND so accurate analyses and predictions can be more reliably made.

**Figure 2:**
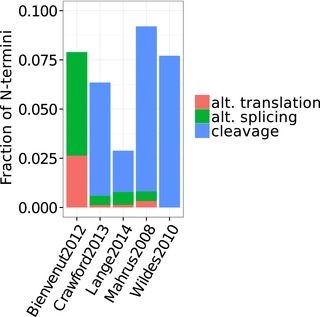
Fraction of explained internal N‐termini in the individual datasets analyzed and the processes identified in each dataset.

The above observations hold true when analyzing individual datasets, as shown in Table 1 and Fig. 2. Less than 10% of internal N‐termini can be explained in any dataset. The explained N‐termini mostly map to known cleavage sites, except for one dataset where N‐termini are analyzed using strong‐cation‐exchange (SCX) chromatography. In this dataset of 38 internal N‐termini, only one N‐terminus could be explained by alternative translation and two N‐termini were explained by alternative splicing (Table 1). While a representative comparison is thus lacking larger numbers of identifications, we suspect a bias against cleavage‐induced N‐termini in SCX data, since SCX focuses on modified N‐termini, which is not the case for other terminomics techniques that identify termini by negative selection in an unbiased manner.

**Table 1:** Internal N‐termini (position > 3) observed and explained in the individual datasets used in this study

Reference	Source	Method	Alternative translation	Alternative splicing	Cleavage	Total
Crawford 2013 24	Cell lines	Subtiligase	9	35	426	7402
Lange 2014 7	Erythrocytes	N‐TAILS	1	5	16	763
Mahrus 2008 25	Cell lines	Subtiligase	4	6	103	1228
Van Damme 2010 26	Cell lines	COFRADIC	0	0	0	0
Wildes 2010 19	Blood plasma	Subtiligase	0	0	41	532
Bienvenut 2012 27	Cell lines	SCX	1	2	0	38

## 5 Protease cleavage and the protease web

Protein cleavage and chain truncation has historically been underestimated, mostly because proteases were considered as enzymes involved principally in degrading proteins and not as precise proteolytic processing devices 18. However, the large extent of protein truncation observed is not surprising considering the hundreds to thousands of new substrates identified for many proteases in relatively few but recent protease substrate screens using TAILS 4, COFRADIC 5, and subtiligase biotinylation 19. Hence, it is becoming increasingly apparent that proteases have a much greater role in molding the proteome than previously appreciated. This is also shown by the large number of proteases evolved in human (460) and other organisms 20 and the complexity of protease regulation on genetic and biochemical levels. In particular, a network of proteases was recently discovered that links proteases together in what has come to be known as the protease web 15. In this network, proteases control the activity of other proteases in complex pathways that largely extend well beyond and bridge the classically defined pathways and cascades. Thereby proteases indirectly influence the cleavage of a large number of proteins in addition to their direct substrates. These effects have been studied in details in the proteolytic networks in cancer 21, the activation of the complement system by MMP2 in inflamed murine skin 6 and in peritonitis by MMP12 22, the interplay between fibrinolysis and complement system 23, and between MMPs and neutrophil serine proteases 15, but can be expected to greatly surpass these detailed examples in the future.

## 6 New software tools

TopFINDer, a new open access tool in TopFIND (http://clipserve.clip.ubc.ca/topfind/topfinder), queries the database for a list of input termini simultaneously and compiles a report that contains termini evidences, general protein and protein domain information, as well as protease statistics for the termini 9. Thereby, TopFINDer accelerates the analysis of terminomics data from days to a few minutes.

To address the challenges in protease substrate assignment posed by the complexity of the protease web the open access software tool PathFINDer (http://clipserve.clip.ubc.ca/topfind/pathfinder) takes the cleavage and inhibition information from TopFIND and queries the protease web for paths between a query protease and a set of input cleavage sites (for example a list of termini from TopFINDer). The resulting paths are sequences of cleavage and inhibition interactions between proteases and their inhibitors that provide insight as to which intermediate inhibitors and proteases might have been indirectly involved in generating the protein truncations identified when studying a protease. Taking all identified paths together, PathFINDer ultimately reports an experiment‐specific network summarizing all identifying paths. These paths and the involved intermediate proteases and inhibitors represent valuable new avenues for hypothesis generation. Thereby, the workings of the network can be validated by targeting intermediate proteins biochemically by chemical or genetic inhibition or knock‐down as reported 15.

## 7 Conclusions

Our present analysis using TopFIND v3.0 has assessed the state of knowledge of the N‐terminome. The pervasiveness of N‐terminal truncations throughout the proteome highlights the importance of both seeking and understanding the effect of protein truncations and the processes generating neo‐termini. This will benefit both an in‐depth understanding of protein and cell biology as well as for the development of targeted therapeutics that avoid clinically relevant drug side effects from unrelated off targets and family member counter targets or anti‐targets. With the complexity of phenotypes of cells and organisms, complex patterns of deep regulation are expected to be at work. However, it remains to be investigated which of the mechanisms of regulation at play have the greatest impact on the phenotype. In addition, truncation of proteins could also be the reason why certain proteins and their peptides are not observed by mass spectrometry. Knowledge of these alternative N‐termini could increase the detection of proteins with proteomics screens using archetypical peptides, SRMs or antibodies. Indeed, if antibodies or SRMs are deployed to target parts of a protein from only the analysis of the sequence, e.g. N‐ or C‐terminal sequence stretches, without considering known cleavage or translation sites, this will lead to systematic false negative results if these regions are present in some conditions but proteolytically removed in others. Therefore, in such cases, the presence and absence of proteins cannot be reliably assessed without experimental knowledge of the population of N‐ and C‐termini of proteins.

To understand and probe complex networks of regulation in the future, data will need agglomeration to then generate specific hypothesis for experimentation. PathFINDer is one such new tool to assist in these efforts. However, the network so far only considers a subset of protease cleavage and inhibition associations and does not yet incorporate kinetic information. In the future we anticipate further refinements of the model to provide more specific and biological meaningful analyses with higher predictive power.

In summary, we assessed the amount and genesis of protein truncation observed experimentally and found that it has a great impact on the majority of proteins in the proteome. Protein truncation is a special protein modification in that it is irreversible and so sets proteins on a path of no return. As compared to alternate translation and splicing, which are also irreversible, protein processing by proteases has very immediate consequence to the phenotype. Thus, we expect protein cleavage to be a quick response to stimuli and for secreted proteins, this is one of the last opportunities for a cell to modify a protein as leaves the cells’ realm of influence.

*The authors have declared no conflict of interest*.

## References

[1] Gaudet, P. , Michel, P.‐A. , Zahn‐Zabal, M. , Cusin, I. et al., The neXtProt knowledgebase on human proteins: current status. Nucleic Acids Res. 2015, 43, D764–D770. 25593349 10.1093/nar/gku1178 PMC4383972

[2] Overall, C. M. , Can proteomics fill the gap between genomics and phenotypes? J. Proteomics 2014, 100, 1–2. 24679705 10.1016/j.jprot.2014.02.025

[3] Lange, P. F. , Overall, C. M. , Protein TAILS: when termini tell tales of proteolysis and function. Curr. Opin. Chem. Biol. 2013, 17, 73–82. 23298954 10.1016/j.cbpa.2012.11.025

[4] Kleifeld, O. , Doucet, A. , auf dem Keller, U. , Prudova, A. et al., Isotopic labeling of terminal amines in complex samples identifies protein N‐termini and protease cleavage products. Nat. Biotechnol. 2010, 28, 281–288. 20208520 10.1038/nbt.1611

[5] Gevaert, K. , Goethals, M. , Martens, L. , Van Damme, J. et al., Exploring proteomes and analyzing protein processing by mass spectrometric identification of sorted N‐terminal peptides. Nat. Biotechnol. 2003, 21, 566–569. 12665801 10.1038/nbt810

[6] auf dem Keller, U. , Prudova, A. , Eckhard, U. , Fingleton, B. , Overall, C. M. , Systems‐level analysis of proteolytic events in increased vascular permeability and complement activation in skin inflammation. Sci. Signal. 2013, 6, rs2. 23322905 10.1126/scisignal.2003512 PMC3872078

[7] Lange, P. F. , Huesgen, P. F. , Nguyen, K. , Overall, C. M. , Annotating N termini for the Human Proteome Project: N termini and Nα‐acetylation status differentiate stable cleaved protein species from degradation remnants in the human erythrocyte proteome. J. Proteome Res. 2014, 13, 2028–2044. 24555563 10.1021/pr401191w PMC3979129

[8] Prudova, A. , Serrano, K. , Eckhard, U. , Fortelny, N. et al., TAILS N‐terminomics of human platelets reveals pervasive metalloproteinase dependent proteolytic processing in storage. Blood 2014, 124, e49–e60. 25331112 10.1182/blood-2014-04-569640 PMC4271184

[9] Fortelny, N. , Yang, S. , Pavlidis, P. , Lange, P. F. , Overall, C. M. , Proteome TopFIND 3.0 with TopFINDer and PathFINDer: database and analysis tools for the association of protein termini to pre‐ and post‐translational events. Nucleic Acids Res. 2015, 43, D290–D297. 25332401 10.1093/nar/gku1012 PMC4383881

[10] Wan, J. , Qian, S.‐B. , TISdb: a database for alternative translation initiation in mammalian cells. Nucleic Acids Res. 2014, 42, D845–850. 24203712 10.1093/nar/gkt1085 PMC3965020

[11] Flicek, P. , Amode, M. R. , Barrell, D. , Beal, K. et al., Ensembl 2014. Nucleic Acids Res. 2014, 42, D749–D755. 24316576 10.1093/nar/gkt1196 PMC3964975

[12] Lange, P. F. , Overall, C. M. , TopFIND, a knowledgebase linking protein termini with function. Nat. Methods 2011, 8, 703–704. 21822272 10.1038/nmeth.1669

[13] Damme, P. V. , Gawron, D. , Criekinge, W. V. , Menschaert, G. , N‐terminal proteomics and ribosome profiling provide a comprehensive view of the alternative translation initiation landscape in mice and men. Mol. Cell. Proteomics 2014, 13, 1245–1261. 24623590 10.1074/mcp.M113.036442 PMC4014282

[14] Gawron, D. , Gevaert, K. , Van Damme, P. , The proteome under translational control. Proteomics 2014, 14, 2647–2662. 25263132 10.1002/pmic.201400165

[15] Fortelny, N. , Cox, J. H. , Kappelhoff, R. , Starr, A. E. , et al., Network analyses reveal pervasive functional regulation between proteases in the Human Protease Web. PLoS Biol. 2014, 12, e1001869. 24865846 10.1371/journal.pbio.1001869 PMC4035269

[16] Gillis, J. , Ballouz, S. , Pavlidis, P. , Bias tradeoffs in the creation and analysis of protein–protein interaction networks. J. Proteomics 2014, 100, 44–54. 24480284 10.1016/j.jprot.2014.01.020 PMC3972268

[17] Rawlings, N. D. , Barrett, A. J. , Bateman, A. , MEROPS: the database of proteolytic enzymes, their substrates and inhibitors. Nucleic Acids Res. 2012, 40, D343–D350. 22086950 10.1093/nar/gkr987 PMC3245014

[18] Turk, B. , Targeting proteases: successes, failures and future prospects. Nat. Rev. Drug Discov. 2006, 5, 785–799. 16955069 10.1038/nrd2092

[19] Wildes, D. , Wells, J. A. , Sampling the N‐terminal proteome of human blood. Proc. Natl. Acad. Sci. 2010, 107, 4561–4566. 20173099 10.1073/pnas.0914495107 PMC2842036

[20] Puente, X. S. , Sánchez, L. M. , Overall, C. M. , López‐Otín, C. , Human and mouse proteases: a comparative genomic approach. Nat. Rev. Genet. 2003, 4, 544–558. 12838346 10.1038/nrg1111

[21] Mason, S. D. , Joyce, J. A. , Proteolytic networks in cancer. Trends Cell Biol. 2011, 21, 228–237. 21232958 10.1016/j.tcb.2010.12.002 PMC3840715

[22] Bellac, C. L. , Dufour, A. , Krisinger, M. J. , Loonchanta, A. , et al., Macrophage matrix metalloproteinase‐12 dampens inflammation and neutrophil influx in arthritis. Cell Rep. 2014, 9, 618–632. 25310974 10.1016/j.celrep.2014.09.006

[23] Krisinger, M. J. , Goebeler, V. , Lu, Z. , Meixner, S. C. et al., Thrombin generates previously unidentified C5 products that support the terminal complement activation pathway. Blood 2012, 120, 1717–1725. 22802338 10.1182/blood-2012-02-412080

[24] Crawford, E. D. , Seaman, J. E. , Agard, N. , Hsu, G. W. , et al., The DegraBase: a database of proteolysis in healthy and apoptotic human cells. Mol. Cell. Proteomics MCP 2013, 12, 813–824. 23264352 10.1074/mcp.O112.024372 PMC3591672

[25] Mahrus, S. , Trinidad, J. C. , Barkan, D. T. , Sali, A. et al., Global sequencing of proteolytic cleavage sites in apoptosis by specific labeling of protein N termini. Cell 2008, 134, 866–876. 18722006 10.1016/j.cell.2008.08.012 PMC2566540

[26] Van Damme, P. , Staes, A. , Bronsoms, S. , Helsens, K. et al., Complementary positional proteomics for screening substrates of endo‐ and exoproteases. Nat. Methods 2010, 7, 512–515. 20526345 10.1038/nmeth.1469

[27] Bienvenut, W. V. , Sumpton, D. , Martinez, A. , Lilla, S. , et al., Comparative large scale characterization of plant versus mammal proteins reveals similar and idiosyncratic N‐α‐acetylation features. Mol. Cell. Proteomics 2012, 11, M111.015131. 22223895 10.1074/mcp.M111.015131 PMC3433923

